# Aquatic plant wax hydrogen and carbon isotopes in Greenland lakes record shifts in methane cycling during past Holocene warming

**DOI:** 10.1126/sciadv.adh9704

**Published:** 2023-09-29

**Authors:** Jamie M. McFarlin, Yarrow Axford, Stephanie Kusch, Andrew L. Masterson, G. Everett Lasher, Magdalena R. Osburn

**Affiliations:** ^1^ Department of Geology and Geophysics, University of Wyoming, Laramie, WY, USA.; ^2^ Department of Earth and Planetary Sciences, Northwestern University, Evanston, IL, USA.; ^3^ Institut des Sciences de la Mer, Université du Québec à Rimouski, Rimouski, Canada.

## Abstract

Predicting changes to methane cycling in Arctic lakes is of global concern in a warming world but records constraining lake methane dynamics with past warming are rare. Here, we demonstrate that the hydrogen isotopic composition (δ^2^H) of mid-chain waxes derived from aquatic moss clearly decouples from precipitation during past Holocene warmth and instead records incorporation of methane in plant biomass. Trends in δ^2^H_moss_ and δ^13^C_moss_ values point to widespread Middle Holocene (11,700 to 4200 years ago) shifts in lake methane cycling across Greenland during millennia of elevated summer temperatures, heightened productivity, and lowered hypolimnetic oxygen. These data reveal ongoing warming may lead to increases in methane-derived C in many Arctic lakes, including lakes where methane is not a major component of the C cycle today. This work highlights a previously unrecognized mechanism influencing δ^2^H values of mid-chain wax and draws attention to the unquantified role of common aquatic mosses as a potentially important sink of lake methane across the Arctic.

## Teaser

δ^2^H values of aquatic plant wax reveal past Holocene warming led to expanded CH_4_ cycling in lakes across Greenland.

## INTRODUCTION

Lakes are a notable natural source of the potent greenhouse gas methane (CH_4_) (*1*). Arctic and boreal landscapes are warming faster than any other region on Earth (*2*) and have the highest density of lakes in the world (*3*). CH_4_ dynamics in high-latitude lakes are sensitive to temperature (*1*, *4*), and warming-driven feedbacks are expected to augment CH_4_ emissions over the coming century (*1*, *5*, *6*). However, forecasting these emissions remains challenging (*7*). Incomplete accounting for increased CH_4_ from Arctic lakes contributes to the substantial uncertainty in projecting the global radiative budget over the coming century (*2*).

Geologic records of insolation-driven Arctic warmth and its consequences in the Early to Middle Holocene (11,700 to 4200 years ago) provide unique opportunities to observe long-term shifts in lake systems in response to past sustained warming. The contributions from Arctic lakes to the Holocene CH_4_ budget are poorly delineated relative to those from tropical and boreal wetlands (*8*, *9*). The C and H isotopic compositions (δ^13^C and δ^2^H, respectively) of atmospheric CH_4_ preserved in ice cores point toward increasing CH_4_ emissions from Arctic lakes during the Middle Holocene, despite overall low global CH_4_ emissions (*9*, *10*). However, past Holocene CH_4_ dynamics in northern lakes are not well constrained (*11*–*13*); thus, limited support is available to test this hypothesis. Here, we present evidence that during the Middle Holocene, the stable isotopic compositions of plant waxes derived from aquatic mosses in Greenland lakes decouple from the hydrological cycle and instead are strongly influenced by uptake of isotopically light (i.e., ^2^H- and ^13^C-depleted, respectively) H and C derived from CH_4_ (*14*). This finding indicates Middle Holocene–aged major changes in CH_4_ dynamics in lakes across Greenland, particularly during the summer when these aquatic mosses grow the most.

Plant wax is composed of *n*-alkyl lipids that are broadly source specific by carbon chain length, with mid-chain (*n*-C_20_-C_25_) compounds dominant in aquatic and nonvascular plant wax and long-chain (*n*-C_26+_) compounds most abundant in terrestrial and vascular plant wax (*15*). These compounds are well preserved in sedimentary records on geologic time scales that range from hundreds of thousands to millions of years (*16*). Waxes extracted from both modern plants and sediments demonstrate that the δ^2^H values of these compounds relate to δ^2^H values of local meteoric water on a global scale, including at Arctic sites (*16*, *17*). This relationship occurs because plant intracellular water derived from local precipitation is predictably modified during lipid biosynthesis, producing waxes with δ^2^H values that are consistently lower than growth water by ~100 to 150‰ depending on the plant type, compound, and growth conditions (*16*, *17*). This widely documented observation provides the basis for reconstructing local precipitation isotopes from sedimentary plant waxes through time. The corresponding C isotopic composition of plant wax additionally relates to the isotopic composition of the inorganic C used during photosynthesis, the metabolic pathway (e.g., C3 versus C4 plants), and growth conditions including water limitation and temperature (*18*). In waxes derived from terrestrial plants, which use atmospheric CO_2_ for photosynthesis, variability in δ^13^C values of sedimentary wax compounds can reflect differences in regional vegetation (e.g., C3 versus C4) or sensitivity to climate (e.g., aridity) (*18*, *19*). In waxes sourced from submerged aquatic plants, which are largely C3 plants, variability in δ^13^C values of sedimentary wax can relate to changes in the size or isotopic composition of the dissolved inorganic C pool (e.g., dissolved CO_2_, bicarbonate) (*20*, *21*).

Although δ^2^H values of plant wax are best known as a proxy with strong empirical relationships to meteoric water (*17*), Middle Holocene δ^2^H values in moss biomarkers at several widespread sites presented here are too isotopically light to be derived from shifts in environmental water alone, requiring a strongly ^2^H-depleted input to explain the observed values. Independent data from other biomarkers and macrofossils indicate that the Middle Holocene was a period of high productivity and low hypolimnetic oxygen, demonstrating that conditions that yield enhanced methanogenesis occurred contemporaneously with the most depleted δ^2^H_moss_ and δ^13^C_moss_ values (see Results). Given this clear environmental change, we hypothesize that CH_4_-derived light H is incorporated into plant wax through a symbiosis between aquatic mosses and methanotrophic bacteria in low-oxygen settings (*22*, *23*) although the exact mechanism by which this can occur, to our knowledge, is currently unknown. Moss-associated methane oxidation (MAMO) is the well-documented symbiosis whereby CH_4_ is oxidized by methanotrophic bacteria residing within *Sphagnum* and brown mosses in some settings (*22*, *23*). Our evidence supports that CH_4_-derived H overprints the isotopic signal from lake water in these biomarkers during the Middle Holocene at multiple sites across Greenland, making aquatic plant wax δ^2^H values at these sites an unreliable proxy for overall δ^2^H_lakewater_ values but alternatively an indicator of changing CH_4_ dynamics.

**Fig. 1.**
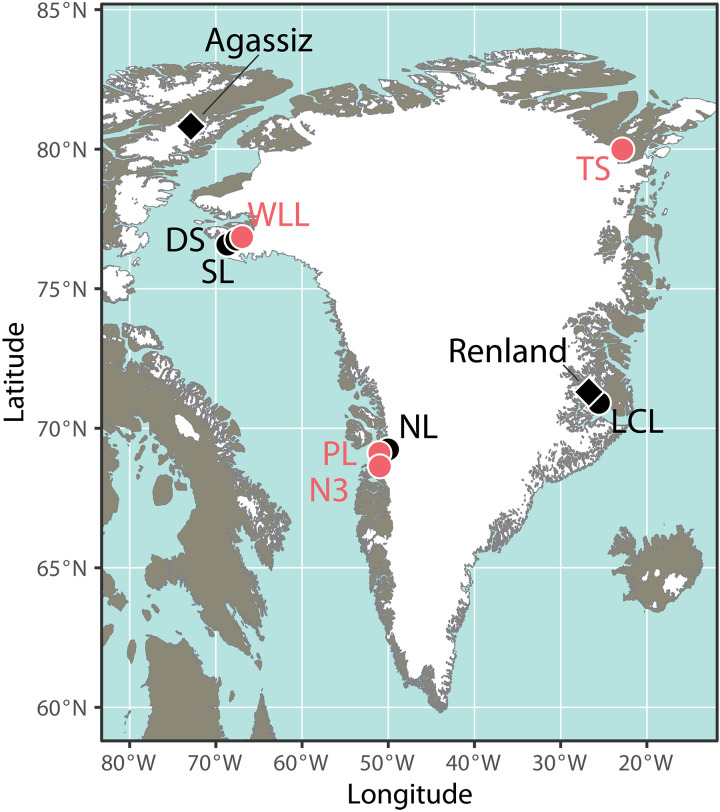
Map of Greenland with sites discussed in text. Red circles are lakes with sedimentary plant wax isotope data included here. Black circles are lakes with published temperature reconstructions referenced in this text. Black diamonds represent locations of ice core records discussed in this text. Lake name abbreviations: WLL, Wax Lips Lake (*25*); TS, Trifna Sø (*26*); N3, Lake N3 (*27*); PL, Pluto Lake (*28*); SL, Secret Lake (*43*); DS, Delta Sø (*80*); NL, North Lake (*29*); LCL, Last Chance Lake (*81*).

**Fig. 2.**
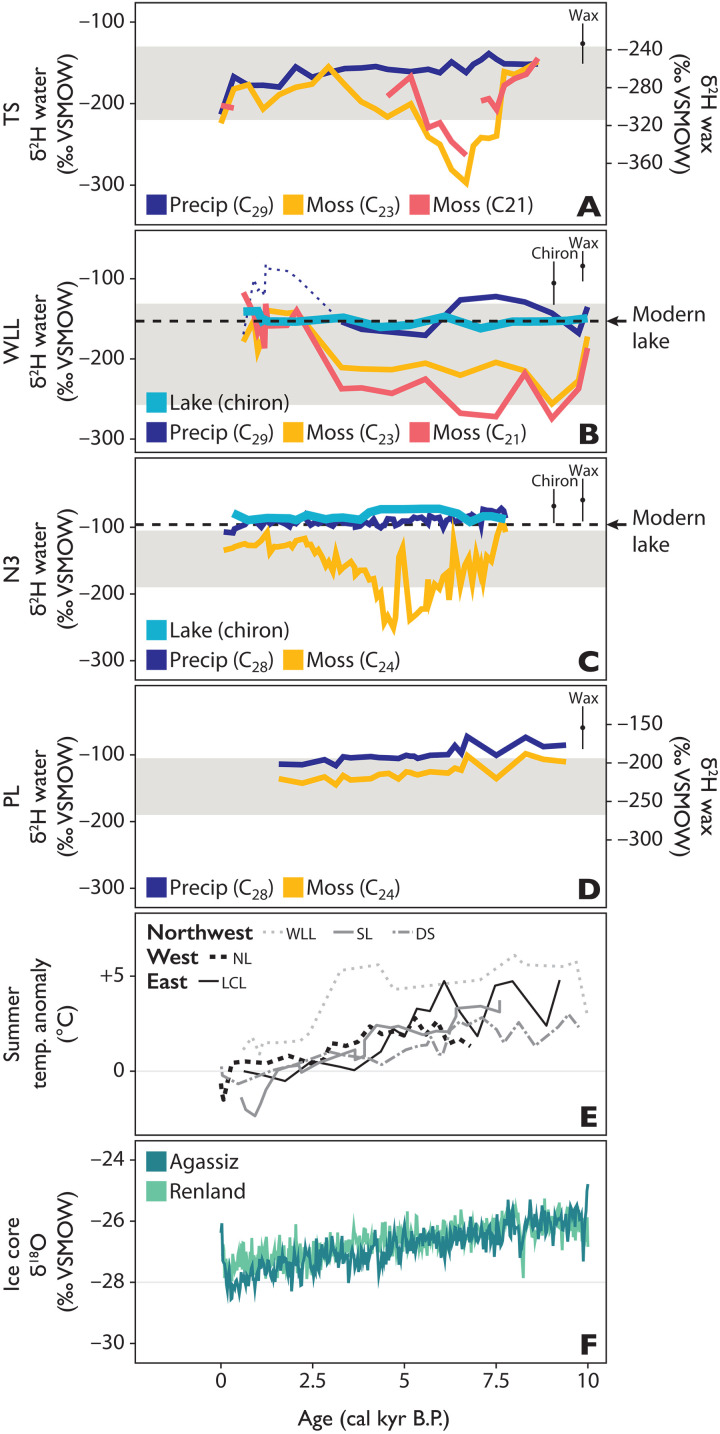
Holocene reconstructed water isotopes and climate. At (**A**) TS, with estimated δ^2^H_precip_ values from long-chain alkanes [C_29_, dark blue, ‰ relative to Vienna Standard Mean Ocean Water (VSMOW)] and estimated δ^2^H_moss_ values from mid-chain alkanes (C_23_, orange and C_21_, red), with additional scaled *y* axis demonstrating raw δ^2^H_wax_ values on right. (**B**) WLL, with estimated δ^2^H_lakewater_ values from δ^18^O_chiron_ (bright blue), estimated δ^2^H_precip_ values from long-chain alkanes (C_29_, dark blue) and estimated δ^2^H_moss_ values from mid-chain alkanes (C_23_, orange and C_21_, red). (**C**) N3, with estimated δ^2^H_lakewater_ values from δ^18^O_chiron_ (bright blue), estimated δ^2^H_precip_ values from long-chain alkanoic acids (C_28_, dark blue) and estimated δ^2^H_moss_ values from mid-chain alkanoic acids (C_24_, orange) (*27*, *28*, *38*). (**D**) PL, with estimated δ^2^H_precip_ values from long-chain alkanoic acids (C_28_, dark blue) and estimated δ^2^H_moss_ values from mid-chain alkanoic acids (C_24_, orange), with additional scaled *y* axis demonstrating raw δ^2^H_wax_ values on right (*28*). (**E**) Summer air temperature anomalies relative to 20th century (pre-1950) from lakes on northwest (WLL, SL, and DS), west (NL), and east Greenland (LCL) (*25*, *29*, *43*, *80*, *81*). (**F**) δ^18^O_ice_ values from the nearby Agassiz (dark green) and Renland (light green) ice caps (*31*, *32*). Light gray bands in (A) to (D) encompass the range of modern precipitation isotopes at each site (upper bound, most ^2^H-enriched summer month average value; lower bound, most ^2^H-depleted winter month average value) estimated using the Online Isotopes of Precipitation Calculator (OIPC) (*35*). Dashed black line in (B) and (C) shows the measured modern δ^2^H_lakewater_ value. Error bars in upper right of (A) to (D) represent the average point propagated error for estimates on wax and chironomid water isotope reconstructions, respectively, including 1σ error from the calibration data and analytical error on the measurements. cal kyr B.P., calibrated thousand years before the present.

We reconstruct plant growth-water isotopes using stable isotope data from aquatic and terrestrial plant wax in four nonglacial lakes on Greenland spanning ~15° of latitude and mean annual temperatures that range from ~−15°C (north Greenland) and ~−10°C (northwest Greenland) to ~−4°C (west Greenland) (Fig. 1) (*24*). To do this, we present new Holocene wax δ^2^H values from Wax Lips Lake (WLL) (*25*) and Trifna Sø (TS) (*26*) and revisit data previously published from Lake N3 (N3) (*27*, *28*) and Pluto Lake (PL) (*28*, *29*). Detailed core descriptions and chronologies have been previously published for all lakes (*25*–*29*). The lakes in this compilation are small (<1 km^2^), through flowing, and have been isolated from glacial meltwater since their deglaciation in the Early Holocene, except for a brief discrete glacial period in the Late Holocene at WLL (*25*). Aquatic brown mosses (Class, Bryopsida; Family, Amblystegiaceae) are the major vegetation in many Arctic lakes (*30*) including the lakes presented here: Macrofossils of aquatic brown mosses (e.g., *Warnstorfia* and *Scorpidium*) are abundant in the sediment cores from these sites, and today, these plants form dense mats on the sediment surfaces (*25*–*28*). We estimate past δ^2^H values of precipitation (δ^2^H_precip_) using δ^2^H values of long-chain sedimentary waxes (C_28_-C_29_; sourced from terrestrial plants), calibrated for plant wax-water fractionation during wax synthesis using global average apparent fractionation factors (ɛ_app_ = −121 ± 18‰ for alkanes, −99 ± 32‰ for acids) (*17*). We reconstruct δ^2^H values of aquatic moss growth water (δ^2^H_moss_) using δ^2^H values of mid-chain sedimentary waxes (C_21_-C_24_, sourced from aquatic plants), calibrated for plant wax-water fractionation using the same constant ɛ_app_ factors as for long-chain waxes stated above (*17*) (Materials and Methods). While the error for each fractionation factor is quite large (±18 to 32‰), the magnitude of change in δ^2^H_moss_ values at WLL, TS, and N3 far exceeds the range in error (Fig. 2). The observed range of δ^2^H_moss_ values in the Middle Holocene at TS, WLL, and N3 requires a distinct source of H with an isotopic composition that is substantially more ^2^H depleted than meteoric water in Greenland from any season and that is integrated into aquatic plant waxes but that does not affect terrestrial plant waxes at these sites. We additionally show new data on the carbon isotopic composition of wax derived from aquatic moss (δ^13^C_moss_) in the Middle Holocene at TS that parallel trends in δ^2^H_moss_ values, linking changes in δ^2^H_moss_ values with a contemporaneous change in the lake C cycle.

## RESULTS

### Holocene precipitation isotopes across Greenland

We find, in agreement with prior publications, that δ^2^H_precip_ values inferred from terrestrial plant waxes at TS, N3, and PL gradually decrease through the Holocene (Fig. 2, A, C, and D), following multimillennial trends similar to those of elevation-corrected oxygen isotope values from the Agassiz and Renland ice caps (δ^18^O_ice_; Fig. 2F) (*31*, *32*), and reflect regional precipitation isotopes at each site. At WLL, δ^2^H_precip_ values from terrestrial plant waxes show a brief, anomalous period during the Late Holocene when δ^2^H_precip_ values are ^2^H-enriched compared to the Middle and Early Holocene (Fig. 2B). Given that increasing δ^2^H_precip_ values in the Late Holocene are not apparent in any other water isotope record from Greenland, including from nearby Agassiz, the Late Holocene trend at WLL likely resulted from a localized change in the origin of plant wax (e.g., increased representation of aerially transported wax from lower latitudes where meteoric and thus growth water is ^2^H enriched compared to Greenland) (*17*) and/or a major change in terrestrial plant growing conditions (e.g., strong aridity driving more enriched leaf water δ^2^H values), rather than a change in isotopic composition of precipitation (*33*). The largest amplitude of change in δ^2^H_precip_ values occurs at the northernmost site TS with lower-amplitude trends at N3 and PL. The diminishing amplitude of change in δ^2^H_precip_ values by latitude is consistent with Holocene temperature reconstructions across Greenland, which show the strongest summer warming (up to ~+5°C relative to modern averages) in the northernmost regions (Fig. 2E) (*25*, *34*).

### H isotopes of aquatic plants decouple from precipitation isotopes at TS, WLL, and N3 during the Middle Holocene

**Fig. 3.**
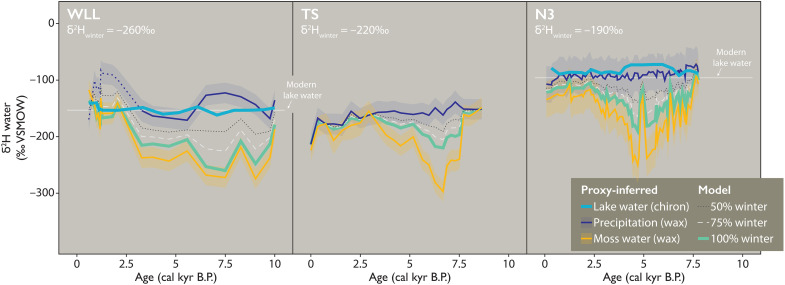
Hydrogen isotope mass balance models for WLL, TS, and N3. Including proxy-inferred δ^2^H_precip_ values (WLL, TS, and N3; dark blue), proxy-inferred δ^2^H_lakewater_ values (WLL and N3; bright blue), proxy-inferred δ^2^H_moss_ values (WLL, TS, and N3; orange), and modeled δ^2^H values of lake water using 100% cold-season endmember (“winter”) precipitation (light green), 75% winter precipitation (dashed, light gray), and 50% winter precipitation (dotted, dark gray) input into the model as maximum contribution to lake water when mid-chain waxes have the lowest δ^2^H value relative to long-chain wax (i.e., the greatest offset between the two proxy values; Materials and Methods). Shading around proxy-inferred δ^2^H_precip_ values (dark blue) and proxy-inferred δ^2^H_moss_ values (orange) represents uncertainty around the inferred value (Materials and Methods).

δ^2^H_moss_ values at TS, WLL, and N3 in the Early-Middle Holocene are substantially more ^2^H depleted than the reconstructed δ^2^H_precip_ values at each site (Fig. 2, A to C). The exact timing and duration of the divergence between δ^2^H_moss_ and δ^2^H_precip_ values vary between TS and N3 (~7.5 to 3 ka) and WLL (~10 to 2.5 ka), but at all three sites, extremely depleted δ^2^H_moss_ values persist for thousands of years until the Late Holocene when δ^2^H_moss_ values shift toward agreement with δ^2^H_precip_ values. Middle Holocene trends in δ^2^H_moss_ values at TS, WLL, and N3 cannot be explained by contemporaneous changes in δ^2^H_precip_ values regionally or seasonally: There is no concurrent ^2^H depletion in terrestrial plant wax in any lake, and moreover, even the most ^2^H-depleted cold-season precipitation (i.e., winter snow) at the highest latitude in Greenland does not have a low enough δ^2^H value to achieve the observed δ^2^H_moss_ values (*35*–*37*) in the Middle Holocene. Expanding on the latter point, we find that δ^2^H_moss_ values are lower than wax-inferred δ^2^H_precip_ values by up to ~160, ~170, and ~180‰ at TS, WLL, and N3, respectively. A mechanism invoked previously to explain divergent H isotope trends in mid- versus long-chain plant waxes, including at N3, is changing seasonality of the precipitation stored in lakes, e.g., increased cold-season precipitation (*27*, *28*, *38*). However, modern seasonal extremes in δ^2^H_precip_ values at each site are too narrow to account for this change, only differing by ~80 to 90‰ at N3 and TS, and ~130‰ in the intensely seasonal climate at WLL (Fig. 2, A to C) (*35*–*37*). We tested this quantitatively with an isotope mass balance model (Fig. 3; Materials and Methods), confirming that this mechanism cannot explain observed Middle Holocene δ^2^H_moss_ values at TS, WLL, and N3. This finding holds even when our model uses climatically improbable conditions (e.g., 100% input of the cold-season endmember, that is, the lowest monthly average δ^2^H_precip_ values observed in modern precipitation, used for δ^2^H_lakewater_ values) and when accounting for analytical uncertainties (i.e., 1σ variability of wax-water fractionation factor): Observed δ^2^H_moss_ values are still at least ~30 to 60‰ lower than the modeled δ^2^H_lakewater_ values at TS and N3 (Fig. 3). As further evidence against a scenario of Middle Holocene winter-dominated δ^2^H_lakewater_ values in Greenland lakes, there is no concurrent signal of increased cold-season precipitation evident in δ^18^O_ice_ values from nearby ice core sites that accumulate precipitation throughout the year. Instead, δ^18^O_ice_ values are highest in the Early-Middle Holocene (Fig. 2F) (*31*, *32*). Furthermore, the between-site similarity of biomarker trends (i.e., the magnitude of the difference in δ^2^H values between terrestrial and aquatic plant waxes along with the timing of their divergence) during the Middle Holocene, especially between TS and N3 despite the distance between these sites and markedly different climate and precipitation regimes (*39*, *40*), indicates that a consistent mechanism is responsible. We also ran more climatically probable models [given that modern mean annual precipitation is slightly warm-season biased at most sites (*24*, *40*)] using 50 and 75% input of cold-season endmember as δ^2^H_lakewater_ values instead: These models yield discrepancies in which observed δ^2^H_moss_ values are lower than modeled δ^2^H_lakewater_ values by up to ~100‰ at both TS and N3 (Fig. 3).

In addition to the above lines of evidence, independent reconstructions of Holocene lake water isotopes at WLL (new data) and N3 (published data) (*38*) are available, based on the oxygen isotopic composition of the chitinous head capsules of obligate aquatic insect larvae [Chironomidae, δ^18^O_chiron_; Materials and Methods; macrofossils from TS were unfortunately not archived after initial counting (*26*) before this work, and thus, isotopic analyses on down-core chironomids cannot be performed for this site]. Chironomid larvae in Arctic lakes live for multiple years and may grow year-round (*41*). Analyses from lakes around the world, including Greenland, and from laboratory cultures show that δ^18^O_chiron_ values are strongly controlled by δ^18^O_lakewater_ values (*42*–*44*). Chironomid δ^18^O values thus provide an independent estimate of the isotopic composition of average lake water to compare against δ^2^H_moss_ and δ^2^H_precip_ values derived from plant wax. The chironomid-inferred δ^18^O_lakewater_ values at both WLL and N3 agree with wax-inferred δ^2^H_precip_ values from terrestrial plant wax from each site (Fig. 2, B and C) and, unlike δ^2^H_moss_ values, do not demonstrate a Middle Holocene period of low values at the two sites where δ^18^O_chiron_ values were measured. In summary, diverse lines of evidence indicate that δ^2^H_moss_ values cannot be explained by changes in the isotopic composition of local meteoric or lake water in the Middle Holocene and another explanation for extremely depleted δ^2^H_moss_ values is required. We reason below that incorporation of H from ^2^H-depleted CH_4_ is the most parsimonious explanation for these Holocene observations in Greenland lakes and is supported by diverse independent evidence.

### Middle Holocene changes in lake stratification, hypolimnetic oxygen, and methane cycling at TS

**Fig. 4.**
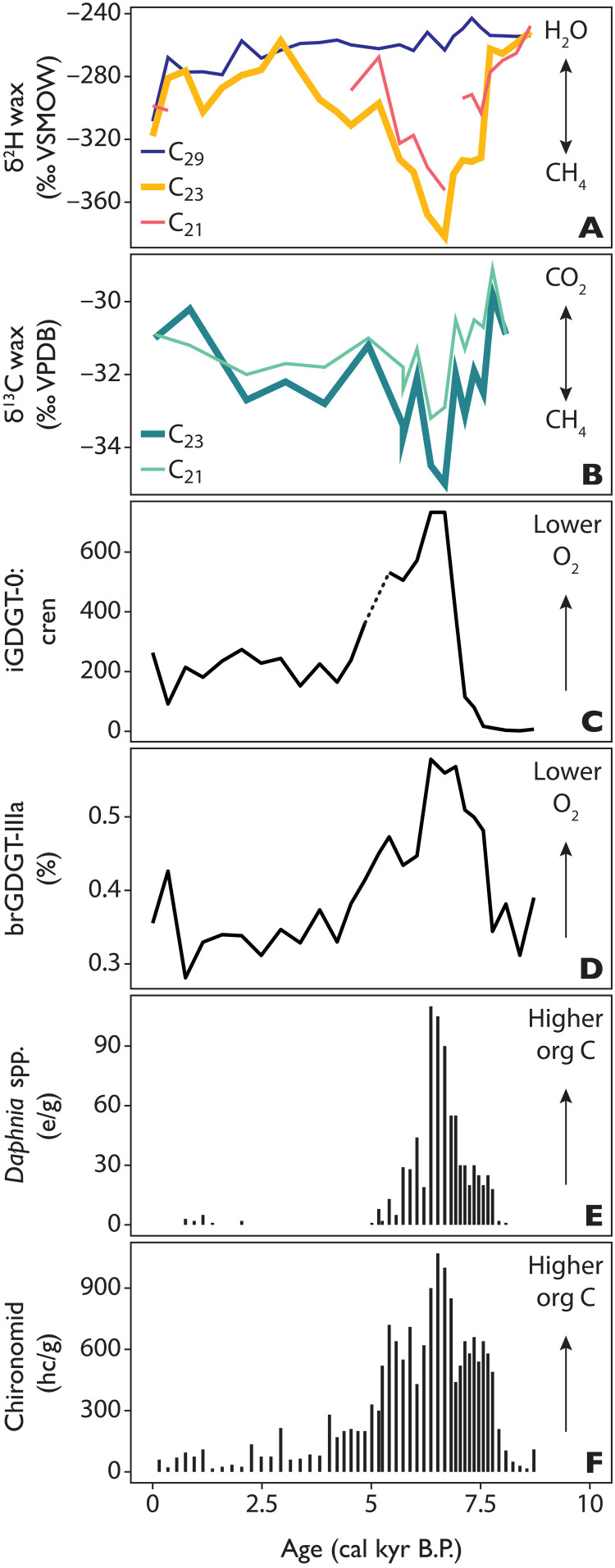
Holocene proxy data from TS. Showing (**A**) δ^2^H_wax_ values of sedimentary long-chain (C_29_, dark blue) and mid-chain alkanes (C_23_, orange and C_21_, red), (**B**) δ^13^C_wax_ values of sedimentary mid-chain alkanes [C_21_, light green and C_23_, dark green, ‰ relative to Vienna Pee Dee Belemnite-LVSEC scale (VPDB)], (**C**) ratio of iGDGT-0 to crenarchaeol (higher values indicate greater methanogenesis and thus lower O_2_), (**D**) fractional abundance of brGDGT IIIa (%) (higher values indicate lower O_2_), concentration of (**E**) aquatic invertebrate remains from *Daphnia* spp. in ephippia per gram dry sediment (e/g) (higher values indicate higher productivity), and (**F**) chironomid larvae in head capsules per gram dry sediment (hc/g) (higher values indicate higher productivity) (*26*).

**Fig. 5.**
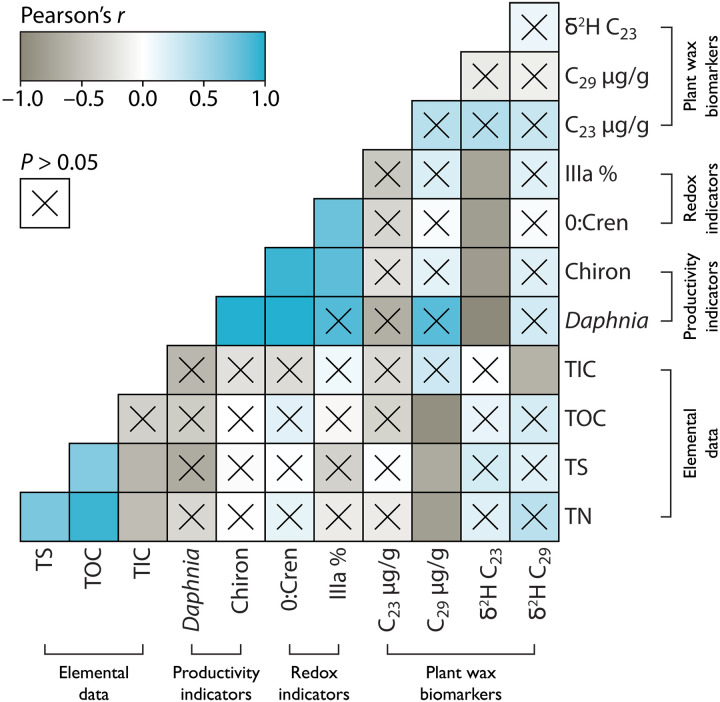
Correlation matrix showing Pearson’s *r* between variables at TS with proxy data grouped by category. Showing elemental data, including total nitrogen (TN), total sulfur (TS), total organic carbon (TOC), and total inorganic carbon (TIC); indicators of general productivity rates, including abundances of ephippia of *Daphnia* spp. carapaces and chironomid head capsules per gram sediment; redox indicators sensitive to hypolimnetic oxygen, including the ratio of iGDGT-0 to crenarchaeol (0:cren), fractional abundances of brGDGT-IIIa as a percent relative to all brGDGT isomers (IIIa %) (*26*); and plant wax measurements, including abundance of C_23_ and C_29_ *n*-alkanes in μg/g TOC, and δ^2^H values of C_23_ and C_29_ *n*-alkanes; where dark brown represents *r* = −1, white represents *r* = 0, bright blue represents *r* = 1, and an “x” over the box indicates the relationship is not significant (*P* > 0.05).

Several proxies, independent of plant wax stable isotope trends, record high carbon loading and intensified lake stratification at TS in northeast Greenland during the Middle Holocene from ~8 to 4 ka (Fig. 4) (*26*). These proxies include macrofossils from aquatic invertebrates and terrestrial plants as well as archaeal and bacterial membrane lipids [i.e., crenarchaeol and isoprenoidal and branched glycerol dialkyl glycerol tetraethers (GDGTs)]. The Middle Holocene at TS is characterized by elevated aquatic invertebrate and terrestrial plant macrofossil abundances, indicating a rise in local productivity both within and around the lake and consequently a greater supply of organic carbon to the lake system (Fig. 4, E and F) (*26*). Increased delivery of organic C to TS is paralleled by evidence for a change in redox conditions toward lower hypolimnetic O_2_ and higher archaeal methanogenesis, demonstrated in the fractional abundance of bacterial branched (br)GDGT-IIIa (*45*) (implying a drop in O_2_), and an increase in the ratio of isoprenoidal (i)GDGT-0:crenarchaeol, (implying strong archaeal methanogenesis) (*46*, *47*) (Fig. 4, C and D). Changing redox conditions indicate a strengthening of stratification—which prevents new dissolved oxygen from the surface layer to be mixed at depth—during the open-water season when peak microbial biomarker production in the Arctic occurs (*48*, *49*). Trends in δ^2^H_moss_ values are highly anticorrelated (*r* = −0.7 to −0.9, *P* < 0.005) to the trends in all these sedimentary indicators but are not related to trends in wax-inferred δ^2^H_precip_ values (Fig. 5). These data support that Holocene changes in δ^2^H_moss_ values are tightly coupled to local changes in productivity and redox conditions within the lake.

**Fig. 6.**
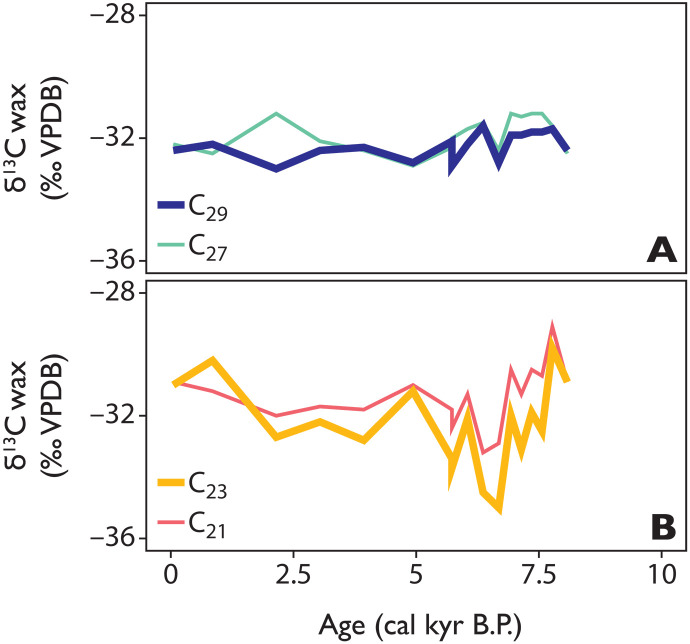
Sedimentary plant wax Holocene carbon isotopic compositions at TS. Including (**A**) long-chain (C_27_, light green and C_29_, dark blue) and (**B**) mid-chain (C_21_, red and C_23_, orange) alkanes from TS (‰ relative to VPDB-LVSEC).

**Fig. 7.**
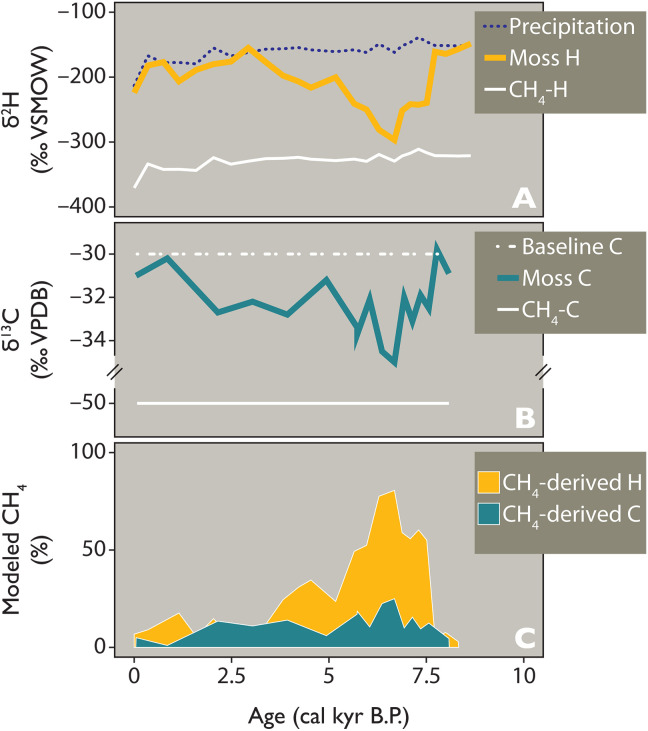
Hydrogen and carbon stable isotope mass balance models for TS. Demonstrating (**A**) proxy-inferred δ^2^H_precip_ values (C_29_ alkane; dotted, dark blue), proxy-inferred δ^2^H_moss_ values (C_23_ alkane, orange), and δ^2^H_CH4_ model values (white); (**B**) δ^13^C_moss_ values (C_23_ alkane, dark green) and δ^13^C model baseline values (dashed, white), and δ^13^C_CH4_ model values (white); and (**C**) modeled % input of CH_4_-derived H (orange) and CH_4_-derived C (dark green) to moss biomass (Materials and Methods).

The stable carbon isotopic composition of sedimentary mid-chain waxes also indicates a major change in the carbon cycle at TS is recorded by aquatic moss biomarkers. At TS, trends in δ^13^C values of sedimentary moss waxes (C_21_ and C_23,_ δ^13^C_moss_) parallel those of δ^2^H_moss_ values, with the most ^13^C-depleted values observed during the Middle Holocene (Fig. 4B), a trend that is again not apparent in terrestrial waxes (Fig. 6). A symbiotic relationship between both *Sphagnum* (Class, Sphagnopsida; Family, Sphagnaceae) and brown mosses and methanotrophic bacteria is well documented (*22*, *23*, *50*). Moss-associated methane-oxidizing bacteria (MOB) form dense colonies within mosses and on moss surfaces (*23*), where they oxidize CH_4_ using O_2_ produced during photosynthesis (*22*, *23*, *51*, *52*). Data from Siberian ponds has shown that 60 to 99% of the CH_4_ produced by sedimentary methanogens in anoxic ponds is oxidized within aquatic brown moss layers (*53*), demonstrating that MAMO is highly efficient at consuming CH_4_ in the water column. Other recent work shows substantial rapid uptake of CH_4_ and N_2_ in submerged mosses with methanotrophic symbionts (*54*). Short-term culturing experiments documenting this symbiosis using ^13^C-labeled CH_4_ have shown that conservatively 30 to 40% of C in new biomass in *Sphagnum* and *Scorpidium*, respectively, is derived from CH_4_ (*22*, *23*). CH_4_-derived C is most strongly incorporated in submerged mosses (compared to nonsubmerged mosses) regardless of the species (*22*, *51*, *52*, *55*). Using a C stable isotope mass balance model, we estimate that during the Middle Holocene, up to ~25% of the C in aquatic moss waxes at TS is derived from CH_4_ (Fig. 7; Materials and Methods), consistent with culturing studies (*22*, *23*). This observation supports both (i) that a change in CH_4_ cycling occurred at TS specifically during the warm-season months when aquatic mosses generate most of their biomass (*30*, *56*, *57*) and (ii) that CH_4_-oxidation products from MAMO including ^13^C-depleted CO_2_ were available to aquatic mosses during the Middle Holocene. Uptake of CH_4_-derived C also provides an intriguing explanation for the strong ^14^C depletion observed for moss fossils in both TS and neighboring Sneha Sø, which thus far had to be explained by reworking (*26*, *58*). MAMO, which can explain relatively depleted δ^13^C_moss_ values at TS during the Middle Holocene, also provides a testable hypothesis to explain how CH_4_-derived H is routed into aquatic moss biomass.

## DISCUSSION

Observations of MAMO in Arctic lakes are limited (*22*) and, to our knowledge, no study has examined whether CH_4_-derived H from MAMO is incorporated into moss biomass, but our findings point to a need for future work in these areas. δ^2^H values of lacustrine CH_4_ are substantially more depleted than δ^2^H values of meteoric water because of strong discrimination against ^2^H during methanogenesis (e.g., δ^2^H_CH4_ values are ~250‰ lower than those of the local growth water available to methanogens in Arctic wetlands) (*14*, *59*). Thus, any CH_4_-derived H in products yielded during methane oxidation (e.g., H_2_O and NH_4_^+^) (*54*) that enter the intracellular H pool of mosses are likely to result in more depleted δ^2^H values of synthesized biomass (*16*). Although biosynthetic pathways by which this can occur in a quantitatively significant manner are currently unclear, we find that when tested in an isotope mass balance model, δ^2^H_moss_ values at WLL, TS, and N3 all require the input of strongly ^2^H-depleted H, consistent with CH_4_-derived H, to achieve the extreme δ^2^H_moss_ values observed during the Middle Holocene (Fig. 7; Materials and Methods).

Using wax-inferred δ^2^H_precip_ values as a baseline for the isotopic composition of contemporaneous lake water at TS, our isotope mass balance model demonstrates that up to ~80% of H in aquatic moss wax at TS comes from a source of H with δ^2^H values 250‰ lower than wax-inferred estimates of average δ^2^H_precip_ values. We reason that the source of this H must be CH_4_ as there are scant other sources of H with such depleted δ^2^H values in lake systems. We note that this is a much higher proportion of CH_4_ than modeled for C, and the specific mechanism for this imbalance also remains unclear. Given our hypothesis that MAMO is providing CH_4_-derived ^13^C-depleted C and ^2^H-depleted H to mosses in these lakes during the Middle Holocene, one possible explanation may reside in the metabolic pathway used by MOB. Limited ^13^C depletion of bacterial biomarkers (−31 to −38‰, in good agreement with our observations) has been demonstrated in *Sphagnum*-associated type II MOB, which use the serine carbon fixation pathway and assimilate CO_2_ in addition to CH_4_ (*60*). Moreover, δ^13^C values in biomarkers from MOB also seem to depend on the ambient temperature, with lower temperatures muting ^13^C fractionation (*61*). Accordingly, the relative amount of MAMO-derived CH_4_-C in the total fixed C pool may be reduced. In addition, strongly ^13^C-enriched CO_2_ produced during methanogenesis may have enriched the lake water δ^13^C values at the time. Regardless, we hypothesize that MAMO is an unexplored route for CH_4_-derived H to be integrated into sedimentary wax isotopes, which are a widely applied paleoenvironmental tool in Arctic lakes. Our isotope mass balance models demonstrate that CH_4_-derived H is needed to explain δ^2^H_moss_ values during the Middle Holocene and must be incorporated into aquatic mosses during this period in some way.

Intracellular water in aquatic plants and mosses is sourced from lake water, and it is thought that methanotrophic symbionts are able to colonize aquatic moss via water transport (*22*, *23*). It is therefore unlikely that the spaces in moss in which methanotrophs reside are not freely exchanging H_2_O. This presents a challenge for identifying the exact mechanism by which MAMO supplies CH_4_-derived H to aquatic mosses in appreciable amounts. If it were limited to the H_2_O produced during the oxidation of CH_4_, then the exchange of CH_4_ would need to be orders of magnitude more rapid than the exchange of water within the cell, given the molarity of lake water relative to the concentration of CH_4_ even in lakes with elevated dissolved CH_4_ (~100 μM CH_4_/liter) (*62*). Prior work has shown that MAMO can oxidize 80 μM CH_4_/g dry plant mass/day (*50*), and there exists speculation that aquatic mosses have unique carbon concentrating mechanisms given how slowly CO_2_ diffuses in water and the inability of these plants to use bicarbonate (*63*). However, to our knowledge, it remains unknown whether this mechanism alone is a sufficient explanation. Understanding the sources of H to NADPH (reduced form of nicotinamide adenine dinucleotide phosphate) pools and the cycling of NADPH in plants is limited but recognized as a major control on the H isotopic composition of intracellular water and subsequently δ^2^H_wax_ values (*16*). In this work, our isotope mass balance model demonstrates that the observed δ^2^H_moss_ values at WLL, TS, and N3 during the Middle Holocene can only be achieved by incorporating CH_4_-derived H. Future studies using modern observations in stratified and methane-producing Arctic lakes with aquatic moss, MAMO culturing experiments, and lipid stable isotope probing are recommended to understand this system in full detail.

### Implications for the impact of past and future warming

Three of the four lakes examined here (TS, WLL, and N3) require the incorporation of CH_4_-derived H into moss wax to fully explain δ^2^H_moss_ values recorded during the Middle Holocene. The fourth lake (PL) does not show extremely depleted δ^2^H_moss_ values and (therefore) appears to not record MAMO. This is consistent with its shallow depth, which would inhibit strong stratification due to wind-driven mixing and thus promote availability of new dissolved O_2_ throughout the open-water season. Only the lakes with moss reported as an abundant constituent in core material and with geometries more prone to thermal stratification in the summer demonstrate uptake of CH_4_-derived H in δ^2^H_moss_ values. CH_4_ production and storage in northern lakes is controlled seasonally by different mechanisms: While many lakes become anoxic during the Arctic winter because of ice cover (*64*), in small lakes (<1-km^2^ surface area) that are relatively deep compared to shallower counterparts and ponds (e.g., >3- to 4-m water depth) and that receive relatively high loads of organic C, CH_4_ concentrations can also increase in the hypolimnion during ice-free summer months when these lakes are thermally stratified and thus O_2_ limited (*65*, *66*). Relatively shallow lakes like PL where the entire water column warms, and those with larger surface areas that promote higher fetch and wind-driven mixing, in contrast are less likely to stratify in the summer. The thickness of the hypolimnion in summer-stratified Arctic lakes increases when light penetration depth decreases as lakes become more productive (*67*) and additionally increases with higher water temperatures and longer ice-free seasons that strengthen thermal stratification (*62*, *66*, *67*). Aquatic moss growth has been shown to strongly favor hypolimnetic water of summer-stratified lakes (*57*), and moss photosynthesis is mostly light and nutrient limited in the Arctic winter; therefore, the conditions that predict MAMO occur specifically during the open-water season in stratified lakes (*30*, *56*, *57*). These requisites align with which lakes in our dataset preserve a signal of CH_4_-derived H in moss biomarkers during the Middle Holocene (TS, WLL, and N3, with maximum water depths of ~6, 9, and 16 m, respectively, and abundant moss noted in sediment cores), and why PL, the shallowest lake in this dataset (water depth of ≤4 m) did not (i.e., this very shallow lake did not experience the same intensity of stratification or hypolimnetic anoxia as the other three sites).

**Fig. 8.**
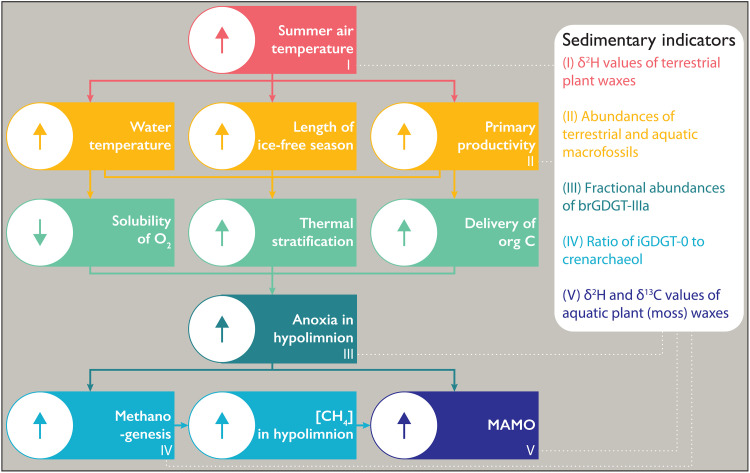
Flow chart of the proposed system changes hypothesized for TS, WLL, and N3 driven by warming air temperatures in the Early-Middle Holocene. Dotted white lines extending from roman numerals identify areas where proxy data from TS provide a sedimentary indicator of the proposed change, with those indicators listed in the panel on the right. Within flow chart, up arrow symbols to the left denote an increase or strengthening of variable, and down arrow symbols denote a decrease or weakening of variable. [CH_4_], concentration of methane.

Precipitation and lake water isotopes at all sites, as inferred from δ^2^H values of long-chain terrestrial plant wax and δ^18^O values of chironomids, respectively, parallel multimillennial trends in Northern Hemisphere polar ice cores and follow the broad, insolation-driven pattern of long-term cooling through the Holocene. Although peak summer warmth occurred in the Early Holocene across much of Greenland (see Fig. 2E), summer temperatures remained elevated above those of the 20th century through the Middle Holocene. Primary production at many sites peaked during the warm Middle Holocene following several millennia of postglacial ecosystem succession and watershed evolution (*34*, *68*). We find that the Middle Holocene combination of warmth and elevated primary production, as previously inferred from aquatic and terrestrial species abundances at TS and many Greenland lakes, was accompanied by major shifts in lake CH_4_ cycling in multiple sectors of Greenland as evident in δ^2^H_moss_ values at WLL, TS, and N3. The observation that δ^2^H_wax_ values of aquatic mosses appear to integrate CH_4_-derived H presents a paradigm shift for interpreting sedimentary records of δ^2^H_wax_ values. Mechanistically, we postulate (i) that a combination of higher summer air and water temperatures and the attendant longer ice-free seasons paired with greater primary production contributed to more persistent summer stratification of TS, WLL, and N3 during the Middle Holocene, when solubility of O_2_ was also lower and delivery of nutrients and organic matter to lakes was higher, and (ii) that these redox conditions promoted methanogenesis, increased CH_4_ cycling in the hypolimnion, and favored MAMO (Fig. 8). The major shifts in lake CH_4_ dynamics at TS, WLL, and N3 occurred during a period of only +1° to 3°C summer warming relative to the 20th century but followed a prolonged Early Holocene period of warming and watershed ontogeny after regional deglaciation.

Strong incorporation of CH_4_-derived H into aquatic moss biomass at TS, WLL, and N3 during the Middle Holocene demonstrates that CH_4_ dynamics drastically changed in widespread lakes for thousands of years during the last period of prolonged relative warmth and greening across Greenland. Elevated methanogenesis in these Arctic lakes occurred when global CH_4_ production was at a Holocene low and may help explain why CH_4_ emissions decreased more strongly in the tropics than the Arctic in the Middle Holocene, despite a reduction in methanogenesis from boreal peatlands (*10*). Incorporation of CH_4_-derived H into aquatic moss biomass, and thus inferred warm-season methanogenesis, decreased at all three affected study sites in response to summer cooling of ~1° to 3°C in the Late Holocene, revealing an important climate dependence on Arctic lake CH_4_ cycling. This paleolimnological perspective from a suite of widespread and climatically diverse Greenland lakes indicates that ongoing warming and extension of the ice-free season, paired with predicted increases in primary production, will drive changes to lake CH_4_ cycling in lakes across Greenland and the Arctic. This, in turn, could lead to higher emissions potential over the coming decades to centuries. Our results also suggest that the widespread presence of aquatic mosses in Arctic lakes may act as a quantitatively important sink for CH_4_—pointing to a complex role for Arctic lakes in the future global carbon cycle. The incorporation of CH_4_-derived H into plant biomass offers a means for reconstructing how lake redox conditions and carbon cycling changed during past periods of sustained warmth.

## MATERIALS AND METHODS

### Sedimentary wax concentration and δ^2^H and δ^13^C values

TS sediment cores are thoroughly described by Kusch *et al*. (*26*), including sediment storage, preparation, and lipid and macrofossil extraction and analyses. WLL sediment cores are thoroughly described by McFarlin *et al.* (*25*). For biomarker work, WLL cores were refrigerated at 4°C for ~1 year before subsampling for biomarker analyses. Lipids were extracted from 0.5 to 3 g of lyophilized sediment using a MARS Microwave Extractor in 20 ml of 9:1 dichloromethane (DCM):MeOH. The extraction program included a 5-min ramp to 100°C, 20 min at 100°C, and a minimum of 30-min cooldown period. Total lipid extracts (TLEs) were filtered and saponified in 0.5 M NaOH at 70°C for 8 to 12 hours and then acidified and separated from the aqueous phase using methyl-tert-butyl ether three times. TLEs were then separated into fractions (alkanes, alcohols, and acids) using Discovery (Sigma-Aldrich) amino-propyl solid-phase extraction (SPE) columns sequentially eluting with hexane, 9:1 DCM:acetone, and 2.5% formate in DCM, respectively. Saturated alkanes were separated from unsaturated alkanes using Discovery (Sigma-Aldrich) Ag-ion SPE columns, eluting with hexane and acetone, respectively.

WLL alkanes were quantified via gas chromatography using a Thermo Fisher Scientific Trace 1310 gas chromatograph (GC) with a ZB5 30-m by 0.25-mm inside diameter (ID) by 0.25-μm film thickness (Zebron) column coupled to flame ionization detector (FID) and a Thermo DSQ single quadrupole mass spectrometer. The GC program used for quantification ramped oven temperatures at 6°C/min from 100° to 330°C. Alkanes were identified via diagnostic mass spectra, comparison to the NIST and in-house libraries, and retention times relative to laboratory standard compounds. Concentrations were calculated from FID peak areas through comparison to that of a 10-μg palmitic acid isobutyl ester internal quantification standard.

Compound-specific carbon (^13^C/^12^C) and hydrogen (^2^H/^1^H) isotopic analyses on alkanes for TS and/or WLL were conducted via GC isotope ratio mass spectrometry using a Thermo Fisher Scientific Trace GC with a ZB5-5MS 30-m by 0.25-mm ID by 1-μm film thickness column coupled to a Thermo Delta V Plus isotope ratio mass spectrometer (IRMS) via a pyrolysis (P) or combustion (C) interface and controlled by a Thermo GC-C III via Isodat. Reactions for GC-pyrolysis-compound specific isotope analysis (GC-P-CSIA) occurred in an alumina column held at 1420°C with a flow of 1.4 ml/min. Reactions for GC-combustion-CSIA (GC-C-CSIA) occurred in an oxidizing reactor consisting of a 2× Cu/Ni/Pt 0.1-m wire bundle held at 940°C in an alumina column. Tank calibration to the VSMOW and VPDB-LSVEC scales used a C_16_-C_30_ *n*-alkane standard (A6, Arndt Schimmelmann, Indiana University) and a derivatized C_14_-C_20_ fatty acid methyl ester (FAME) mixed standard (F8b, Alex Sessions, Caltech). Instrumental error for GC-P-CSIA was assessed via root mean SE on F8 and A6, which was run between every three sample duplicates and averages <5‰ (alkanes). The average analytical error (1σ) for GC-C-CSIA is 0.15‰ VPDB-LSVEC. The H_3_^+^ factor was determined and applied regularly and averaged 5.214 ppm/nA during the analytical period during 2016 when WLL samples were run and averaged 3.394 ppm/nA during the analytical period during 2019 when TS samples were run. For each sample, the alkane fractions were measured in duplicate for ^2^H/^1^H- and ^13^C/^12^C-isotope analyses. Compound-specific δ^2^H and δ^13^C values are reported as average duplicate values on the VSMOW and VPDB-LSVEC scale, respectively. Total analytical error presented on δ^2^H and δ^13^C values includes the 1σ of measurements on each compound peak as well as the instrumental error over the course of all sample measurements, propagated using the root sum of squares. δ^13^C values of FAMEs were corrected for the addition of the methyl group during derivatization via a derivatized phthalic acid of known C isotopic composition.

### Oxygen isotopic composition of chironomid head capsules

WLL sediment samples of 4 to 8 ccs (representing 2 cm of core depth) were collected for chironomid δ^18^O analysis. Each sample was deflocculated in a 10% KOH solution at 20°C for 30 min and then sieved in deionized water using 150-μm mesh. Chironomid head capsules were manually picked and cleaned of any remaining adhering material under a dissecting microscope and then transferred to oven dried 3.2-mm by 4-mm lightweight Elemental Microanalysis silver capsules. On average, ~100 head capsules were collected for each analysis (totaling ~100 μg of analyte material). The filled silver capsules were thoroughly freeze dried and analyzed for δ^18^O values on a Thermo Fisher Scientific High Temperature Conversion Elemental Analyzer (TC/EA) coupled to a Thermo Fisher Scientific Delta V IRMS via a Conflo IV interface in the Northwestern University Stable Isotope Laboratory. The TC/EA pyrolysis was conducted at 1450°C, and standardization to the VSMOW scale was done via calibration with a mixture of organic (e.g., benzoic acid) and inorganic (e.g., BaSO_4_) standards including benzoic acid #A (Indiana University) and NBS127, IAEA-SO5, and IAEA-SO6. Average precision on standards during the analytical period was ±0.4‰. Original chironomid δ^18^O values are reported as per mil (‰) relative to VSMOW.

A down-core subset of isolated chironomid remains were also checked for the presence of carbonates using Fourier transform infrared spectroscopy (FT-IR). Individual head capsules were scanned on a Bruker Hyperion 2000 series FT-IR Microscope system between 600 and 4000 cm^−1^ at 32 times per 4-cm^−1^ resolution. Peaks characteristic of carbonate at ∼712, ∼862, and ∼1440 cm^−1^ [corresponding to the in-plane bending vibration, the out-of-plane bending vibration, and the asymmetric stretching of bonds in a CO_3_^2−^ molecule (*69*–*71*)] were not present in the spectra of any samples.

Analysis of down-core chironomid δ^18^O values were attempted for TS, but not enough chironomid material remained to perform isotope measurements. Unfortunately, chironomid samples originally prepared by Kusch *et al.* (*26*) were not archived.

### Correction of H wax and O chironomid isotope values to water isotope values

The δ^2^H values of plant wax alkanes and acids are calibrated to water δ^2^H values using the global average apparent fractionation factor estimated from *n*-C_29_ alkanes (ɛ_app_ = −121 ± 18‰) and *n*-C_28_ alkanoic acids (ɛ_app_ = −99 ± 32‰) respectively from McFarlin *et al.* (*17*). The 1σ error on each calibration is represented with error bars in the upper right corner in Fig. 2 (A to D). These calibrations are estimated from sedimentary long-chain waxes but applied to both mid- and long-chain compounds here because there is existing precedent in the wax literature to assume wax-water fractionation factors of aquatic plants are equal to that of terrestrial plants (*27*, *28*, *72*–*74*). This precedent is supported by the available observations of wax-water fractionation factors in sedimentary waxes that show statistically indistinguishable fractionation factors between mid-chain waxes and lake water [ɛ_app_ = −125 ± 36‰, *n* = 44 for *n*-C_23_ alkanes and ɛ_app_ = −112 ± 33‰, *n* = 110 for *n*-C_24_ alkanoic acids in global data compiled by McFarlin *et al.* (*17*)] compared to long-chain waxes and precipitation, albeit with substantially fewer observations of the former. This precedent is also supported by agreement within error of the apparent fractionation values observed in modern aquatic and nonvascular plants (*17*, *75*–*77*). While larger fractionation effects have been observed in some aquatic plants growing in saline water or heterotrophically in dark conditions (*78*, *79*), neither of these parameters are consistent with aquatic moss growth during the Arctic summer in nonglacial freshwater lakes (*30*, *57*).

The δ^18^O values of chironomid head capsules are converted to δ^18^O_lakewater_ values using a regression from van Hardenbroek *et al.* (*42*) (Eq. 1) with residual SE on this estimate of 2.4‰. δ^2^H_WLL_ values are estimated from δ^18^O_lakewater_ values using the local meteoric water line (LMWL), which is constrained using observations recorded in the IAEA-GNIP database from the nearby Pitiffuk Station (Thule, ~60 km from WLL) (Eq. 2) (*36*), with residual SE on this relationship of 9‰. δ^2^H_N3_ values are estimated from δ^18^O_lakewater_ values using the LMWL for western Greenland reported by Corcoran *et al.* (Eq. 3) (*38*). The error on chironomid-estimated δ^2^H_lakewater_ values represented in Fig. 2 for WLL and N3 is compounded error that includes the analytical error on the oxygen isotope measurements of chironomids (±0.4‰ for WLL and ±0.2‰ for N3) (*38*), residual SE on the van Hardenbroek *et al.* (*42*) regression (±2.4‰), both of which are propagated during O to H isotope calculations, and residual SE on the LMWL (±9‰, estimated using the LWML from WLL).

$$\delta^{18}O_{\mathrm{lakewater}}(\text{‰}\mathrm{VSMOW})=(0.96^{*}\delta^{18}O_{\mathrm{chiron}})-22.6$$ (1)

$$\delta^{2}H_{\mathrm{WLL}}(\text{‰}\mathrm{VSMOW})=(7.33^{*}\delta^{18}O_{\mathrm{lakewater}})-7.1$$ (2)

$$\delta^{2}H_{N3}(\text{‰}\mathrm{VSMOW})=(6.8^{*}\delta^{18}O_{\mathrm{lakewater}})-13.7$$ (3)

### Water H isotope mass-balance model for TS, WLL, and N3

We assume that δ^2^H_precip_ values, estimated from δ^2^H values of long-chain plant waxes (*n*-C_29_ alkanes at TS, WLL; *n*-C_28_ acids at N3), are representative of the growth water available to terrestrial plants during the summer growing season at each site and that this precipitation will also enter the lake. δ^2^H_precip_ values therefore provide one input to lake water at each site and can be used to assess whether endmember δ^2^H_winter_ values (i.e., the most ^2^H-depleted winter precipitation isotopes observed in modern monthly data, estimated at −190‰, −220‰, and −260‰ at N3, TS, and WLL, respectively) (*35*, *37*) can fully explain the observed δ^2^H_moss_ values at their lowest point in the Middle Holocene (Eq. 4). We assume that where δ^2^H_moss_ values are the lowest value (i.e., largest offset) relative to δ^2^H_precip_ values (ɛ_max_) during the Middle Holocene represents when moss growth water (i.e., δ^2^H_lakewater_) would have been most weighted toward winter precipitation if this were indeed the mechanism driving trends in δ^2^H_moss_ values at each site and allow the models to incorporate 100% endmember δ^2^H_winter_ values for δ^2^H_lakewater_ (0% wax-inferred δ^2^H_precip_ values) at this point in time for each record. We then scale the input of endmember δ^2^H_winter_ values at all other points in time relative to this point based on the epsilon value between mid- and long-chain wax δ^2^H values (ɛ_terr-aq_) (Eq. 5) given the assumption being tested here that differences in ɛ_terr-aq_ throughout the Holocene are a function of the amount of winter input to each lake.

$$\delta^{2}H_{\mathrm{lakewater}}(\text{‰}\mathrm{VSMOW})=(n)*\delta^{2}H_{\mathrm{winter}}+(1-n)*\delta^{2}H_{\mathrm{precip}}$$ (4)

$$n=\varepsilon_{\mathrm{terr}-\mathrm{aq}}/\varepsilon_{\max}$$ (5)

Because of the uncertainty in the apparent fractionation factor used to calibrate δ^2^H_wax_ values to δ^2^H_precip_ or δ^2^H_moss_ values, we also allow for a 1σ error in these estimates, which is represented in Fig. 3. Because there is no incorporation of δ^2^H_precip_ values at the point when δ^2^H_moss_ values are the lowest, the estimates of wax-inferred δ^2^H_precip_ values are somewhat irrelevant to testing whether any amount of winter precipitation can fully explain light δ^2^H_moss_ values. We find that 100% winter endmember precipitation cannot explain δ^2^H_moss_ values at TS or N3 during the Middle Holocene and that 100% winter endmember precipitation can only just explain the Early to Middle Holocene trends at WLL if we apply the largest fractionation factor in our range (ɛ_app_ = −139‰). However, full incorporation of only an extreme winter endmember is climatically improbable (e.g., even an average value of winter precipitation across all winter months would be substantially ^2^H enriched relative to the winter endmember), and we have also included models that set the maximum uptake of winter endmember precipitation at 75 and 50% (Eqs. 6 and 7).

$$n_{75}=0.75^{*}(\varepsilon_{\mathrm{terr}-\mathrm{aq}}/\varepsilon_{\max})$$ (6)

$$n_{50}=0.50^{*}(\varepsilon_{\mathrm{terr}-\mathrm{aq}}/\varepsilon_{\max})$$ (7)

These latter models are more climatically probable and yield lake water values that are in closer agreement with the H isotopic composition of mean annual precipitation at each site (*35*, *37*). We note that modern lake water δ^2^H values at both WLL (−153‰) and N3 (−96‰) are currently strongly summer biased (*28*), demonstrating little retention of winter precipitation in lake water into the summer months at these sites at present. We unfortunately have no measurements of modern lake water isotopes for TS. The δ^2^H_lakewater_ values that result from mixing of the two precipitation endmembers we present here demonstrate the improbability that winter precipitation would ever dominate in these lakes, where typically equal or more precipitation amounts are delivered during the summer season (*24*, *40*) and given the competing input of more enriched summer precipitation during the Early-Middle Holocene at each site.

### CH_4_-isotope mass balance model for TS

We posit that C and H derived from CH_4_ provides an alternative source of exceptionally light H and C isotopes to aquatic plant wax. We estimate the amount of CH_4_-derived H that is used during lipid biosynthesis by aquatic moss by using an isotope mass balance model that assumes δ^2^H_moss_ values are a product of two endmembers: wax-inferred δ^2^H_precip_ values and δ^2^H_CH4_ values as H-derived from CH_4_. δ^2^H_CH4_ values are estimated through the Holocene at TS using the dataset from Chanton *et al.* (*14*), which demonstrates biogenic δ^2^H_CH4_ in high-latitude wetlands is ~250‰ lower than growth water, with environmental water through the Holocene at TS estimated using wax-inferred δ^2^H_precip_ values (Eq. 8). The percent of CH_4_-derived H represented in δ^2^H_moss_ values is estimated using Eq. 9.

$$\delta^{2}H_{\mathrm{CH}4}\;(\text{‰}\mathrm{VSMOW})=1000*\{[(1000+\delta^{2}H_{\mathrm{precip}})/1000-(-250)]-1\}$$ (8)

$${CH}_{4}\text{-}\mathrm{derived}\;H\;(\%)=(\delta^{2}H_{\mathrm{moss}}-\delta^{2}H_{\mathrm{precip}})/(\delta^{2}H_{CH4}-\delta^{2}H_{\mathrm{precip}})$$ (9)

We estimate the amount of CH_4_-derived C that is used during lipid biosynthesis by aquatic moss by using an isotope mass balance model that assumes biologic fractionation during wax synthesis is constant and that δ^13^C_moss_ values are a product of two endmembers: a baseline of δ^13^C_CO2_ = −30‰ (VPDB), which represents the value of δ^13^C_moss_ in the Early Holocene, before the onset of depleted isotope values in the Middle Holocene and assume δ^13^C_CH4_ = −50‰ (VPDB) as a conservative estimate based on modern environmental observations in several West Greenland lakes by Cadieux *et al.* (*66*), which range from ~−30 to −70‰ (VPDB). The percent of CH_4_-derived C represented in δ^13^C_moss_ values at TS is estimated using Eq. 10.

$${\mathrm{CH}}_{4}\text{-}\mathrm{derived}\;C\;(\%)=(\delta^{13}C_{\mathrm{moss}}-\delta^{13}C_{\mathrm{CO}2})/(\delta^{13}C_{\mathrm{CH}4}-\delta^{13}C_{\mathrm{CO}2})$$ (10)

## References

[1] M. Wik, R. K. Varner, K. W. Anthony, S. MacIntyre, D. Bastviken, Climate-sensitive northern lakes and ponds are critical components of methane release. Nat. Geosci. 9, 99–105 (2016).

[2] M. Collins, R. Knutti, J. Arblaster, T. Fichefet, P. Friedlingstein, X. Gao, W. J. Gutowski, T. Johns, G. Krinner, M. Shongwe, C. Tebaldi, A. J. Weaver, M. Wehner, 2013: Long-term Climate Change: Projections, Commitments and Irreversibility, in *Climate Change 2013: The Physical Science Basis. Contribution of Working Group 1 to the Fifth Assessment Report of the Intergovernmental Panel on Climate Change, *T.F. Stocker, D. Qin, G.-K.Plattner, M. Tignor, S.K. Allen, J. Boschung, A. Nauels, Y. Xia, V. Bex and P.M. Midgley, Eds. (Cambridge Univ. Press, 2014), pp. 1029–1136.

[3] C. Verpoorter, T. Kutser, D. A. Seekell, L. J. Tranvik, A global inventory of lakes based on high-resolution satellite imagery. Geophys. Res. Lett. 41, 6396–6402 (2014).

[4] M. Wik, B. F. Thornton, D. Bastviken, S. MacIntyre, R. K. Varner, P. M. Crill, Energy input is primary controller of methane bubbling in subarctic lakes. Geophys. Res. Lett. 41, 555–560 (2014).

[5] K. M. Walter, L. C. Smith, F. S. Chapin, Methane bubbling from northern lakes: Present and future contributions to the global methane budget. Philos. Trans. Royal Soc. A. 365, 1657–1676 (2007). 10.1098/rsta.2007.2036 17513268

[6] K. M. Walter, S. A. Zimov, J. P. Chanton, D. Verbyla, F. S. Chapin, Methane bubbling from Siberian thaw lakes as a positive feedback to climate warming. Nature 443, 71–75 (2006). 16957728 10.1038/nature05040

[7] M. Engram, K. M. Walter Anthony, T. Sachs, K. Kohnert, A. Serafimovich, G. Grosse, F. J. Meyer, Remote sensing northern lake methane ebullition. Nat. Clim. Chang. 10, 511–517 (2020).

[8] Y. Zheng, J. S. Singarayer, P. Cheng, X. Yu, Z. Liu, P. J. Valdes, R. D. Pancost, Holocene variations in peatland methane cycling associated with the Asian summer monsoon system. Nat. Commun. 5, 4631 (2014). 25135106 10.1038/ncomms5631 PMC4143914

[9] Y. Zheng, Z. Fang, T. Fan, Z. Liu, Z. Wang, Q. Li, R. D. Pancost, B. D. A. Naafs, Operation of the boreal peatland methane cycle across the past 16 k.y. Geology 48, 82–86 (2020).

[10] T. Sowers, Atmospheric methane isotope records covering the Holocene period. Quat. Sci. Rev. 29, 213–221 (2010).

[11] H. Sundqvist, D. Kaufman, N. McKay, N. Balascio, J. Briner, L. Cwynar, H. Sejrup, H. Seppa, D. Subetto, J. Andrews, Arctic Holocene proxy climate database–new approaches to assessing geochronological accuracy and encoding climate variables. Clim. Past. 10, 1605–1631 (2014).

[12] M. Elvert, J. W. Pohlman, K. W. Becker, B. Gaglioti, K. U. Hinrichs, M. J. Wooller, Methane turnover and environmental change from Holocene lipid biomarker records in a thermokarst lake in Arctic Alaska. Holocene 26, 1766–1777 (2016).

[13] M. J. Wooller, J. W. Pohlman, B. v. Gaglioti, P. Langdon, M. Jones, K. M. W. Anthony, K. W. Becker, K. U. Hinrichs, M. Elvert, Reconstruction of past methane availability in an Arctic Alaska wetland indicates climate influenced methane release during the past ~12,000 years. J. Paleolimnol. 48, 27–42 (2012).

[14] J. P. Chanton, D. Fields, M. E. Hines, Controls on the hydrogen isotopic composition of biogenic methane from high-latitude terrestrial wetlands. Eur. J. Vasc. Endovasc. Surg. 111, (2006)X.

[15] R. T. Bush, F. A. McInerney, Leaf wax *n*-alkane distributions in and across modern plants: Implications for paleoecology and chemotaxonomy. Geochim. Cosmochim. Acta 117, 161–179 (2013).

[16] D. Sachse, I. Billault, G. J. Bowen, Y. Chikaraishi, T. E. Dawson, S. J. Feakins, K. H. Freeman, C. R. Magill, F. A. McInerney, M. T. J. J. van der Meer, P. Polissar, R. J. Robins, J. P. Sachs, H.-L. Schmidt, A. L. Sessions, J. W. C. White, J. B. West, A. Kahmen, Molecular paleohydrology: Interpreting the hydrogen-isotopic composition of lipid biomarkers from photosynthesizing organisms. Annu. Rev. Earth Planet. Sci. 40, 221–249 (2012).

[17] J. M. McFarlin, Y. Axford, A. L. Masterson, M. R. Osburn, Calibration of modern sedimentary δ2H plant wax-water relationships in Greenland lakes. Quat Sci Rev. 225, 105978 (2019).

[18] A. F. Diefendorf, E. J. Freimuth, Extracting the most from terrestrial plant-derived *n*-alkyl lipids and their carbon isotopes from the sedimentary record: A review. Org. Geochem. 103, 1–21 (2017).

[19] M. J. Kohn, Carbon isotope compositions of terrestrial C3 plants as indicators of (paleo)ecology and (paleo)climate. Proc. Natl. Acad. Sci. 107, 19691–19695 (2010). 21041671 10.1073/pnas.1004933107 PMC2993332

[20] G. D. Farquhar, J. R. Ehleringer, K. T. Hubick, Carbon Isotope discrimination and photosynthesis. Annu. Rev. Plant. Physiol. Plant. Mol. Biol. 40, 503–537 (1989).

[21] B. Aichner, U. Herzschuh, H. Wilkes, Influence of aquatic macrophytes on the stable carbon isotopic signatures of sedimentary organic matter in lakes on the Tibetan Plateau. Org. Geochem. 41, 706–718 (2010).

[22] S. Liebner, J. Zeyer, D. Wagner, C. Schubert, E.-M. Pfeiffer, C. Knoblauch, Methane oxidation associated with submerged brown mosses reduces methane emissions from Siberian polygonal tundra. J. Ecol. 99, 914–922 (2011).

[23] A. A. Raghoebarsing, A. J. P. Smolders, M. C. Schmid, W. I. C. Rijpstra, M. Wolters-Arts, J. Derksen, M. S. M. Jetten, S. Schouten, J. S. Sinninghe Damsté, L. P. M. Lamers, J. G. M. Roelofs, H. J. M. Op den Camp, M. Strous, Methanotrophic symbionts provide carbon for photosynthesis in peat bogs. Nature 436, 1153–1156 (2005). 16121180 10.1038/nature03802

[24] J. Cappelen, C. Drost Jensen, “Climatological Standard Normals 1991–2020--Greenland” (Danish Meteorological Institute, 2021).

[25] J. M. McFarlin, Y. Axford, M. R. Osburn, M. A. Kelly, E. C. Osterberg, L. B. Farnsworth, Pronounced summer warming in northwest Greenland during the Holocene and Last Interglacial. Proc. Natl. Acad. Sci. 115, 6357–6362 (2018). 29866819 10.1073/pnas.1720420115 PMC6016770

[26] S. Kusch, O. Bennike, B. Wagner, M. Lenz, I. Steffen, J. Rethemeyer, Holocene environmental history in high-Arctic North Greenland revealed by a combined biomarker and macrofossil approach. Boreas 48, 273–286 (2019).

[27] E. K. Thomas, J. P. Briner, J. J. Ryan-Henry, Y. Huang, A major increase in winter snowfall during the middle Holocene on western Greenland caused by reduced sea ice in Baffin Bay and the Labrador Sea. Geophys. Res. Lett. 43, 5302–5308 (2016).

[28] E. K. Thomas, K. V. Hollister, A. A. Cluett, M. C. Corcoran, Reconstructing arctic precipitation seasonality using aquatic leaf wax δ^2^H in lakes with contrasting residence times. Paleoceanogr Paleoclimatol. 35, e2020PA003886 (2020).

[29] Y. Axford, S. Losee, J. P. Briner, D. R. Francis, P. G. Langdon, I. R. Walker, Holocene temperature history at the western Greenland Ice Sheet margin reconstructed from lake sediments. Quat Sci Rev. 59, 87–100 (2013).

[30] K. Sand-Jensen, T. Riis, S. Markager, W. F. Vincent, Slow growth and decomposition of mosses in Arctic lakes. Can. J. Fish. Aquat. Sci. 56, 388–393 (1999).

[31] B. S. Lecavalier, D. A. Fisher, G. A. Milne, B. M. Vinther, L. Tarasov, P. Huybrechts, D. Lacelle, B. Main, J. Zheng, J. Bourgeois, A. S. Dyke, High Arctic Holocene temperature record from the Agassiz ice cap and Greenland ice sheet evolution. Proc. Natl. Acad. Sci. U.S.A. 114, 5952–5957 (2017). 28512225 10.1073/pnas.1616287114 PMC5468641

[32] B. M. Vinther, S. L. Buchardt, H. B. Clausen, D. Dahl-Jensen, S. J. Johnsen, D. A. Fisher, R. M. Koerner, D. Raynaud, V. Lipenkov, K. K. Andersen, T. Blunier, S. O. Rasmussen, J. P. Steffensen, A. M. Svensson, Holocene thinning of the Greenland ice sheet. Nature 461, 385–388 (2009). 19759618 10.1038/nature08355

[33] A. Kahmen, B. Hoffmann, E. Schefuß, S. K. Arndt, L. A. Cernusak, J. B. West, D. Sachse, Leaf water deuterium enrichment shapes leaf wax *n*-alkane δD values of angiosperm plants II: Observational evidence and global implications. Geochim. Cosmochim. Acta 111, 50–63 (2013).

[34] Y. Axford, E. C. Osterberg, A. de Vernal, Past warmth and its impacts during the Holocene Thermal Maximum in Greenland. Annu. Rev. Earth Planet. Sci. 49, 279–307 (2021).

[35] G. J. Bowen, The Online Isotopes in Precipitation Calculator, version 3.1 (2020); http://wateriso.utah.edu/waterisotopes/pages/data_access/oipc.html.

[36] International Atomic Energy Agency/World Meteorological Organization, Global Network of Isotopes in Precipitation. The GNIP Database (2020).

[37] G. J. Bowen, L. I. Wassenaar, K. A. Hobson, Global application of stable hydrogen and oxygen isotopes to wildlife forensics. Oecologia 143, 337–348 (2005). 15726429 10.1007/s00442-004-1813-y

[38] M. C. Corcoran, E. K. Thomas, C. Morrill, Using a paired chironomid δ^18^O and aquatic leaf wax δ^2^H approach to reconstruct seasonality on western greenland during the holocene. Paleoceanogr. Paleoclimatol. 36, e2020PA004169 (2021).

[39] E. Hanna, S. H. Mernild, J. Cappelen, K. Steffen, Recent warming in Greenland in a long-term instrumental (1881–2012) climatic context: I. Evaluation of surface air temperature records. Environ. Res. Lett. 7, 045404 (2012).

[40] S. H. Mernild, E. Hanna, J. R. McConnell, M. Sigl, A. P. Beckerman, J. C. Yde, J. Cappelen, J. K. Malmros, K. Steffen, Greenland precipitation trends in a long-term instrumental climate context (1890–2012): Evaluation of coastal and ice core records. Int. J. Climatol. 35, 303–320 (2015).

[41] D. R. Oliver, Life history of the chironomidae. Annu. Rev. Entomol. 16, 211–230 (1971).

[42] M. van Hardenbroek, A. Chakraborty, K. L. Davies, P. Harding, O. Heiri, J. Schilder, C. N. Trueman, M. J. Wooller, The stable isotope composition of organic and inorganic fossils in lake sediment records: Current understanding, challenges, and future directions. Quat Sci Rev. 196, 154–176 (2018).

[43] G. E. Lasher, Y. Axford, J. M. McFarlin, M. A. Kelly, E. C. Osterberg, M. B. Berkelhammer, Holocene temperatures and isotopes of precipitation in Northwest Greenland recorded in lacustrine organic materials. Quat Sci Rev. 170, 45–55 (2017).

[44] Y. V. Wang, D. M. O’Brien, J. Jenson, D. Francis, M. J. Wooller, The influence of diet and water on the stable oxygen and hydrogen isotope composition of Chironomidae (Diptera) with paleoecological implications. Oecologia 160, 225–233 (2009). 19238449 10.1007/s00442-009-1303-3

[45] J. E. Tierney, "GDGT thermometry: Lipid tools for reconstructing paleotemperatures" in *Reconstructing Earth’s Deep-Time Climate: The State of the Art in 2012*, L. C. Ivany, B. T. Huber, Eds. (2012), pp. 115–131.

[46] C. I. Blaga, G.-J. Reichart, O. Heiri, J. S. Sinninghe Damsté, Tetraether membrane lipid distributions in water-column particulate matter and sediments: A study of 47 European lakes along a north–south transect. J. Paleolimnol. 41, 523–540 (2009).

[47] J. Lattaud, C. De Jonge, A. Pearson, F. J. Elling, T. I. Eglinton, Microbial lipid signatures in Arctic deltaic sediments – Insights into methane cycling and climate variability. Org. Geochem. 157, 104242 (2021).

[48] J. Cao, Z. Rao, F. Shi, G. Jia, Ice formation on lake surfaces in winter causes warm-season bias of lacustrine brGDGT temperature estimates. Biogeosciences 17, 2521–2536 (2020).

[49] J. H. Raberg, D. J. Harning, S. E. Crump, G. de Wet, A. Blumm, S. Kopf, Á. Geirsdóttir, G. H. Miller, J. Sepúlveda, Revised fractional abundances and warm-season temperatures substantially improve brGDGT calibrations in lake sediments. Biogeosciences 18, 3579–3603 (2021).

[50] N. Kip, J. F. van Winden, Y. Pan, L. Bodrossy, G.-J. Reichart, A. J. P. Smolders, M. S. M. Jetten, J. S. S. Damsté, H. J. M. Op den Camp, Global prevalence of methane oxidation by symbiotic bacteria in peat-moss ecosystems. Nat. Geosci. 3, 617–621 (2010).

[51] N. Basiliko, R. Knowles, T. R. Moore, Roles of moss species and habitat in methane consumption potential in a northern peatland. Wetlands 24, 178–185 (2004).

[52] R. Zibulski, F. Wesener, H. Wilkes, B. Plessen, L. A. Pestryakova, U. Herzschuh, C / N ratio, stable isotope (δ^13^ C, δ^15^ N), and *n*-alkane patterns of brown mosses along hydrological gradients of low-centred polygons of the Siberian Arctic. Biogeosciences 14, 1617–1630 (2017).

[53] C. Knoblauch, O. Spott, S. Evgrafova, L. Kutzbach, E.-M. Pfeiffer, Regulation of methane production, oxidation, and emission by vascular plants and bryophytes in ponds of the northeast Siberian polygonal tundra. J. Geophys. Res. Biogeosci. 120, 2525–2541 (2015).

[54] T. Larmola, S. M. Leppänen, E.-S. Tuittila, M. Aarva, P. Merila, H. Fritze, M. Tiirola, Methanotrophy induces nitrogen fixation during peatland development. Proc. Natl. Acad. Sci. 111, 734–739 (2014). 24379382 10.1073/pnas.1314284111 PMC3896166

[55] T. Larmola, E.-S. Tuittila, M. Tiirola, H. Nykänen, P. J. Martikainen, K. Yrjälä, T. Tuomivirta, H. Fritze, The role of Sphagnum mosses in the methane cycling of a boreal mire. Ecology 91, 2356–2365 (2010). 20836457 10.1890/09-1343.1

[56] C.-Q. Guo, R. Ochyra, P.-C. Wu, R. D. Seppelt, Y.-F. Yao, L.-G. Bian, S.-P. Li, C.-S. Li, *Warnstorfia exannulata*, an aquatic moss in the Arctic: Seasonal growth responses. Clim. Change 119, 407–419 (2013).

[57] T. Riis, K. Sand-Jensen, Growth reconstruction and photosynthesis of aquatic mosses: Influence of light, temperature and carbon dioxide at depth. J. Ecol. 85, 359–372 (1997).

[58] B. Wagner, O. Bennike, Holocene environmental change in the Skallingen area, eastern North Greenland, based on a lacustrine record. Boreas 44, 45–59 (2015).

[59] L. S. Brosius, K. M. Walter Anthony, G. Grosse, J. P. Chanton, L. M. Farquharson, P. P. Overduin, H. Meyer, Using the deuterium isotope composition of permafrost meltwater to constrain thermokarst lake contributions to atmospheric CH_4_ during the last deglaciation. Eur. J. Vasc. Endovasc. Surg. 117 10.1029/2011JG001810, (2012).

[60] J. F. van Winden, N. Kip, G.-J. Reichart, M. S. M. Jetten, H. J. M. O. den Camp, J. S. S. Damsté, Lipids of symbiotic methane-oxidizing bacteria in peat moss studied using stable carbon isotopic labelling. Org. Geochem. 41, 1040–1044 (2010).

[61] J. F. van Winden, H. M. Talbot, G. Reichart, N. P. McNamara, A. Benthien, J. S. Sinninghe Damsté, Influence of temperature on the δ^13^C values and distribution of methanotroph-related hopanoids in Sphagnum-dominated peat bogs. Geobiology 18, 497–507 (2020). 32180328 10.1111/gbi.12389 PMC7383571

[62] R. M. Northington, J. E. Saros, Factors controlling methane in arctic lakes of Southwest Greenland. PLOS ONE 11, e0159642 (2016). 27454863 10.1371/journal.pone.0159642 PMC4959701

[63] J. Glime, "Nutrient relations: CO_2_" in *Bryophyte Ecology* (2017); http://digitalcommons.mtu.edu/bryophyte-ecology/.

[64] S. B. Cadieux, J. R. White, L. M. Pratt, Exceptional summer warming leads to contrasting outcomes for methane cycling in small Arctic lakes of Greenland. Biogeosciences 14, 559–574 (2017).

[65] S. Juutinen, M. Rantakari, P. Kortelainen, J. T. Huttunen, T. Larmola, J. Alm, J. Silvola, P. J. Martikainen, Methane dynamics in different boreal lake types. Biogeosciences 6, 209–223 (2009).

[66] S. B. Cadieux, J. R. White, P. E. Sauer, Y. Peng, A. E. Goldman, L. M. Pratt, Large fractionations of C and H isotopes related to methane oxidation in Arctic lakes. Geochim. Cosmochim. Acta 187, 141–155 (2016).

[67] J. E. Saros, R. M. Northington, C. L. Osburn, B. T. Burpee, N. John Anderson, Thermal stratification in small arctic lakes of southwest Greenland affected by water transparency and epilimnetic temperatures. Limnol. Oceanogr. 61, 1530–1542 (2016).

[68] S. C. Fritz, N. J. Anderson, The relative influences of climate and catchment processes on Holocene lake development in glaciated regions. J. Paleolimnol. 49, 349–362 (2013).

[69] X. Liu, S. M. Colman, E. T. Brown, E. C. Minor, H. Li, Estimation of carbonate, total organic carbon, and biogenic silica content by FTIR and XRF techniques in lacustrine sediments. J. Paleolimnol. 50, 387–398 (2013).

[70] F. Reig, J. V. G. Adelantado, M. C. M. M. Moreno, FTIR quantitative analysis of calcium carbonate (calcite) and silica (quartz) mixtures using the constant ratio method. Application to geological samples. Talanta 58, 811–821 (2002). 18968811 10.1016/s0039-9140(02)00372-7

[71] M. S. Xia, Z. T. Yao, L. Q. Ge, T. Chen, H. Y. Li, A potential bio-filler: The substitution effect of furfural modified clam shell for carbonate calcium in polypropylene. J. Compos. Mater. 49, 807–816 (2015).

[72] O. Rach, A. Kahmen, A. Brauer, D. Sachse, A dual-biomarker approach for quantification of changes in relative humidity from sedimentary lipid *D*/*H* ratios. Clim. Past 13, 741–757 (2017).

[73] N. L. Balascio, W. J. D’Andrea, R. S. Bradley, B. B. Perren, Biogeochemical evidence for hydrologic changes during the Holocene in a lake sediment record from southeast Greenland. Holocene 23, 1428–1439 (2013).

[74] L. Curtin, W. J. D’Andrea, N. Balascio, G. Pugsley, G. de Wet, R. Bradley, Holocene and Last Interglacial climate of the Faroe Islands from sedimentary plant wax hydrogen and carbon isotopes. Quat. Sci. Rev. 223, 105930 (2019).

[75] N. L. Balascio, W. J. D’Andrea, R. S. Anderson, S. Wickler, Influence of vegetation type on *n*-alkane composition and hydrogen isotope values from a high latitude ombrotrophic bog. Org. Geochem. 121, 48–57 (2018).

[76] H. Dion-Kirschner, J. M. McFarlin, A. L. Masterson, Y. Axford, M. R. Osburn, Modern constraints on the sources and climate signals recorded by sedimentary plant waxes in west Greenland. Geochim. Cosmochim. Acta 286, 336–354 (2020).

[77] B. Aichner, U. Herzschuh, H. Wilkes, A. Vieth, J. Böhner, δD values of *n*-alkanes in Tibetan lake sediments and aquatic macrophytes – A surface sediment study and application to a 16ka record from Lake Koucha. Org. Geochem. 41, 779–790 (2010).

[78] D. Yakir, M. J. DeNiro, Oxygen and hydrogen isotope fractionation during cellulose metabolism in Lemna gibba L. Plant Physiol. 93, 325–332 (1990). 16667454 10.1104/pp.93.1.325 PMC1062506

[79] B. Aichner, S. Hilt, C. Périllon, M. Gillefalk, D. Sachse, Biosynthetic hydrogen isotopic fractionation factors during lipid synthesis in submerged aquatic macrophytes: Effect of groundwater discharge and salinity. Org. Geochem. 113, 10–16 (2017).

[80] Y. Axford, G. E. Lasher, M. A. Kelly, E. C. Osterberg, J. Landis, G. C. Schellinger, A. Pfeiffer, E. Thompson, D. R. Francis, Holocene temperature history of northwest Greenland – With new ice cap constraints and chironomid assemblages from Deltasø. Quat Sci Rev. 215, 160–172 (2019).

[81] Y. Axford, L. B. Levy, M. A. Kelly, D. R. Francis, B. L. Hall, P. G. Langdon, T. V. Lowell, Timing and magnitude of early to middle Holocene warming in East Greenland inferred from chironomids. Boreas 46, 678–687 (2017).

